# The role of interleukin-8 (IL-8) and IL-8 receptors in platinum response in high grade serous ovarian carcinoma

**DOI:** 10.18632/oncotarget.3415

**Published:** 2015-03-23

**Authors:** Euan A. Stronach, Paula Cunnea, Christina Turner, Tankut Guney, Radhika Aiyappa, Senthuran Jeyapalan, Camila H. de Sousa, Alacoque Browne, Nesreen Magdy, James B. Studd, Ruethairat Sriraksa, Hani Gabra, Mona El-Bahrawy

**Affiliations:** ^1^ Ovarian Cancer Action Research Centre, Department of Surgery and Cancer, Imperial College London, London, UK; ^2^ Department of Histopathology, Imperial College London, Hammersmith Hospital, London, UK; ^3^ Division of Molecular Medicine, St. John's Research Institute, Bangalore, India; ^4^ Department of Pathology, National Cancer Institute, Cairo University, Egypt; ^5^ Epigenetics Group, International Agency for Research on Cancer, Lyon, France; ^6^ Department of Pathology, Faculty of Medicine, University of Alexandria, Egypt

**Keywords:** ovary, carcinoma, cytokine, interleukin-8, chemoresistance

## Abstract

Platinum based drugs are the cornerstone of chemotherapy for ovarian cancer, however the development of chemoresistance hinders its success. IL-8 is involved in regulating several pro-survival pathways in cancer. We studied the expression of IL-8 and IL-8 receptors in platinum sensitive and resistant cell lines. Using qRT-PCR and immunohistochemistry, both platinum sensitive (PEA1, PEO14) and resistant (PEA2, PEO23) show increased expression of IL-8 and IL-8 receptors. IL-8RA shows nuclear and cytoplasmic expression, whilst IL-8RB is present solely in the cytoplasm. Knockdown of IL-8 increased sensitivity to cisplatin in platinum sensitive and reversed platinum resistance in resistant cell lines, decreased the expression of anti-apoptotic Bcl-2 and decreased inhibitory phosphorylation of pro-apoptotic Bad. IL-8 receptor antagonist treatment also enhanced platinum sensitivity. Nuclear localisation of IL-8RA was only detected in platinum resistant tumours. Inhibition of IL-8 signalling can enhance response in platinum sensitive and resistant disease. Nuclear IL-8RA may have potential as a biomarker of resistant disease.

## INTRODUCTION

The principal treatment strategy for most cases of ovarian carcinoma is primary cytoreductive surgery and platinum-based chemotherapy, usually using cisplatin or carboplatin along with a taxane (e.g paclitaxel). Approximately 75% of patients who are initially responsive to chemotherapy can develop platinum resistance [1], with a poor outcome for patients that is both due to the silent progressive nature of ovarian cancer and the development of resistance to chemotherapy [2–4]. Among the causes of acquired drug resistance in ovarian cancer are secretion of molecules that confer resistance [5] and limitation of factors required for cancer cell death [6]. The immune system is implicated in ovarian cancer progression [7] and may also be involved in the acquisition of drug resistance via the cytokine and chemokine signalling pathways [8].

Interleukin-8 (IL-8) is a pro-inflammatory chemokine that is principally a chemoattractant and activator of neutrophils during an immune response [9]. It also has cell growth, angiogenic and motogenic effects in different types of malignancies including melanoma, ovarian, prostate, and colon carcinoma [10, 11]. The levels of IL-8 are elevated in ovarian cyst fluid, ascites, serum, and tumour tissue from patients with ovarian cancer [12]. Overexpression of IL-8 in ovarian cancer cells increases anchorage-independent growth, proliferation, angiogenic potential, adhesion and invasion. These effects are decreased on depletion of endogenous IL-8 expression by transfecting IL-8-overexpressing SKOV-3 cells with plasmid encoding for antisense IL-8 [13]. Studies have shown that high expression of IL-8 in ovarian cancer patients is significantly correlated with poor prognosis [14, 15], with advanced tumour stage and high tumour grade [16].

Experimental studies in prostate carcinoma highlighted that IL-8 expression confers chemoresistance [17]. Links between resistance to chemotherapeutic agents and IL-8 in ovarian carcinoma have been previously demonstrated. A paclitaxel-resistant ovarian cancer cell line, SKOV-3TR, overexpressed a set of genes which included *IL-8*, suggesting that the development of paclitaxel resistance is accompanied by multiple changes in gene expression including stable alterations in selective chemokine and cytokine expression [18]. Also previous studies have shown that paclitaxel can induce *IL-8* gene expression [19, 20].

The aim of this study was to explore the role of IL-8 in platinum response in ovarian carcinoma. We show the relationship between platinum treatment and expression of IL-8 and IL-8 receptors and the relation to platinum resistance in ovarian high grade serous carcinoma (HGS) both *in vitro* and in clinical samples.

## RESULTS

### Expression of IL-8 and IL-8 receptors in cell lines

**Figure 1:**
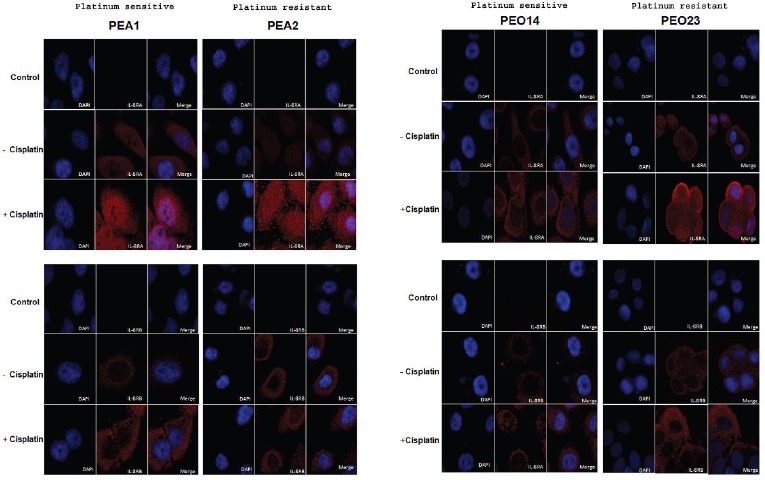
Expression of IL-8RA and IL-8RB in ovarian cancer cells pre and post cisplatin treatment Expression levels increase on cisplatin treatment and are generally higher in resistant as compared to sensitive cell lines. Images show cytoplasmic and nuclear expression of IL-8RA and cytoplasmic expression of IL-8RB. (Control: negative control; no incubation with primary antibody)

To investigate the *in vitro* expression and sub-cellular localisation of IL-8RA and IL-8RB protein levels, and the effect of cisplatin treatment on receptor expression, sensitive and resistant ovarian high-grade serous cancer cells were subjected to cisplatin treatment for 24 h and then studied by immunofluorescence and confocal microscopy. The intensity of expression and localization of receptors were also investigated under untreated conditions. The results confirm expression of IL-8 receptors in all cell lines tested. The level of receptor expression is generally higher in the resistant lines. Nuclear localisation of IL-8RA was observed on cisplatin treatment in all cell lines (Figure 1).

**Figure 2:**
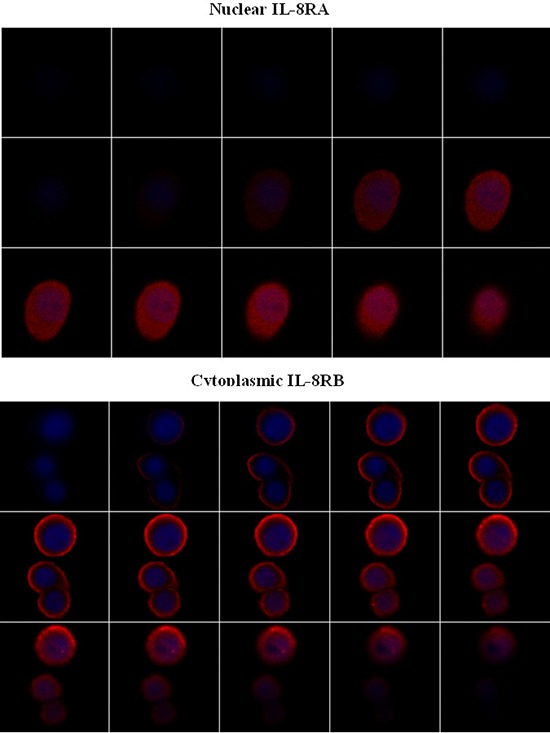
Subcellular localisation of IL-8 receptors on cisplatin treatment Z stack confirms nuclear expression of IL-8RA and only cytoplasmic expression of IL-8RB.

Examination of the cells at multiple levels using confocal microscopy confirms the presence of IL-8RA in the nucleus of cells post cisplatin treatment, in contrast to IL-8RB, which shows no nuclear localisation. A series of optical sections confirms these findings (Figure 2).

**Figure 3:**
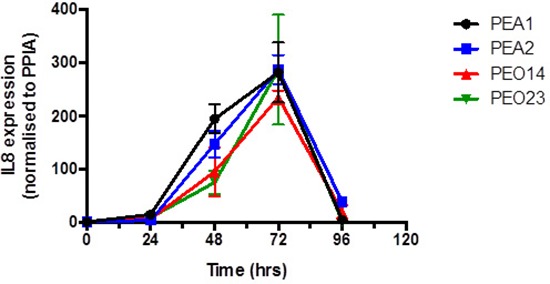
Expression of IL-8 on platinum treatment in isogenic pairs of platinum sensitive and platinum resistant cell lines There is a consistent trend of increased *IL-8* expression up to a peak at 72 hours followed by drop to basal levels around 96 hours in both platinum sensitive and resistant ovarian cancer cells.

All cell lines show expression of *IL-8* with a rapid transcriptional induction of *IL-8* following platinum exposure, reaching a maximum expression peak of over 200 fold above baseline following 72 hours exposure, reducing back to near basal levels at 96 hours post platinum treatment (Figure 3). *IL-8* mRNA levels increase more rapidly in PEA1 and PEA2 than in PEO14 and PEO23. At 48 hours post cisplatin treatment, PEA1 and PEA2 cell lines undergo 150–200 fold induction of *IL-8* levels compared to PEO14 and PEO23 which show 95 and 75 fold induction at 48 hours (Figure 3).

### Effect of IL-8 pathway inhibition on cell sensitivity to cisplatin

IL-8 was knocked down in the intra-patient paired platinum sensitive and resistant cell lines, PEA1/PEA2 and PEO14/PEO23. Knockdown of *IL-8* was confirmed by qRT-PCR in all lines.

**Figure 4:**
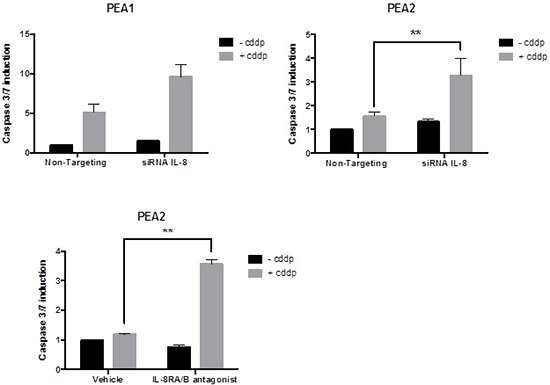
A. The effect of siRNA for IL-8 on platinum sensitivity siRNA for IL-8 increases platinum sensitivity in PEA1 and reverses platinum resistance in PEA2. **B.** The effect of blocking IL-8 Signalling using an IL-8RA/RB receptor antagonist: Blocking IL-8 receptors reverses platinum resistance in PEA2 cells. (cddp = cisplatin) The vertical axis represents fold change in apoptosis.

All cell lines showed a reduction in *IL-8* expression in siRNA treated cells of > 50% when compared to untreated controls. Following cisplatin treatment IL-8 siRNA treated cells show enhanced apoptotic response to platinum in PEA1 (clinically platinum sensitive, ∼2 fold; not statistically significant) and PEA2 (clinically platinum resistant, > 2 fold; *P* = 0.0018) cells (Figure 4A).

We sought to further confirm that blocking IL-8 signalling influences chemoresistance, using an IL-8 receptor A/B antagonist. Blocking of IL-8 A/B receptors re-sensitised PEA2 cells to cisplatin treatment (25 μm), as shown by a significant increase in apoptosis (> 3-fold; *P* = 0.0041) compared to the cisplatin only treated cells (Figure 4B). This observation mirrors the results obtained by siRNA knockdown of IL-8 compared to the non-targeting control (Figure 4A).

### The relationship between IL-8 signalling, response to platinum treatment and pro-apoptotic proteins

Previous studies have shown that anti-apoptotic protein levels are constitutively higher in platinum resistant cells. To understand the mechanism of enhancement of platinum response following IL-8 pathway inhibition, we studied the levels of two key regulators of apoptosis, Bcl-2 and pBad-S136 following IL-8 signalling blockage by siIL-8 treatment.

**Figure 5:**
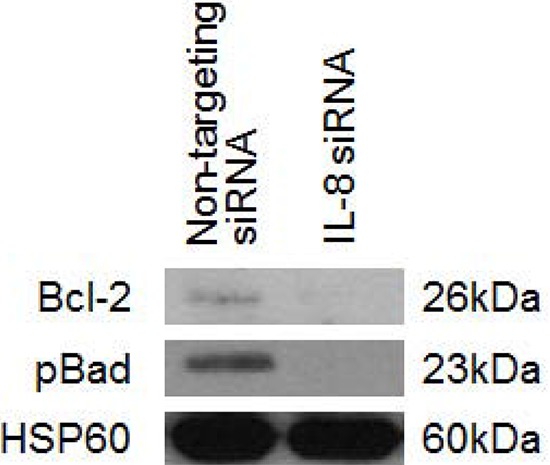
The effect of IL-8 knockdown on the expression levels of anti-apoptotic proteins IL-8 knockdown by siRNA causes reduction of expression of total Bcl-2 protein and in phosphorylation of Bad at serine residue 136. Both of these events are associated with pro-apoptotic signalling. (cddp = cisplatin)

Western blot analysis indicates that knockdown of IL-8 causes noticeable reduction of anti-apoptotic Bcl-2 protein (Figure 5). This observation is mirrored by the reduction of inhibitory Bad phosphorylation at S136 following knockdown of IL-8 compared to the non-targeting control (Figure 5). Loss of anti-apoptotic proteins after IL-8 knockdown and cisplatin treatment explains the increase in apoptosis indicated by the elevation of caspase activity after IL-8 knockdown and drug treatment (Figure 4).

### Expression of IL-8 receptors in ovarian high grade serous carcinomas

**Figure 6:**
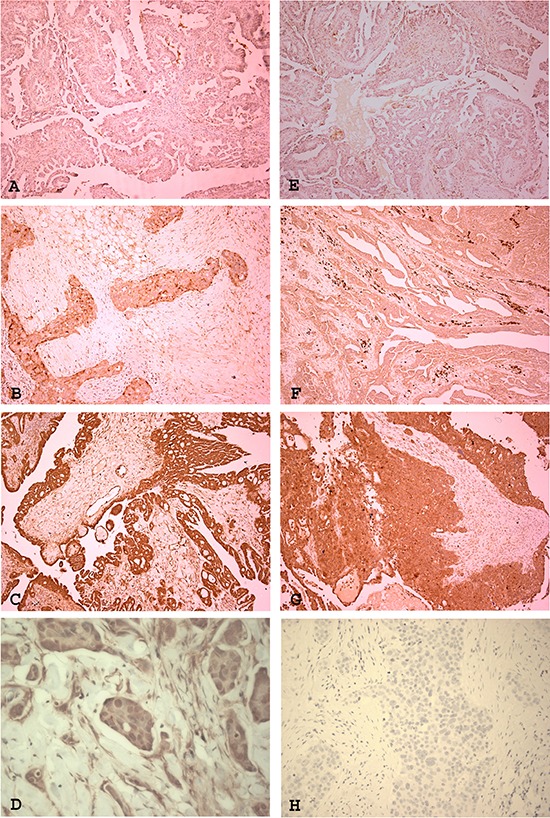
Expression of IL-8 receptors RA and RB in ovarian high grade serous carcinoma The receptors are expressed at variable intensity in tumours. IL-8RA showed weak **(A)**, moderate **(B)** and strong expression [magnification ×100]. Some cases showed nuclear localisation of IL-8RA **(D)** [magnification ×400]. IL-8RB showed weak **(E)**, moderate **(F)** and strong **(G)** expression [magnification ×100]. **(H)** Negative control [magnification ×400]

In order to investigate if our *in vitro* findings reflect clinical expression of receptors, we studied the expression and subcellular distribution of IL-8RA and IL-8RB by immunohistochemistry in 41 high grade serous ovarian tumours, which included platinum sensitive (*n* = 17) and resistant tumours (*n* = 24). Expression was seen at variable intensity in the majority of tumours (Figure 6). The levels of expression of IL-8RA and IL-8RB are presented in tables 1 and 2 respectively. No statistically significant correlation was found between the level of expression of either receptor and platinum sensitivity, however, nuclear localisation of IL-8RA was seen in 4 cases, all of which were platinum resistant (Figure 6D).

**Table 1:** expression of IL-8RA in ovarian high grade serous carcinoma

	Resistant	Sensitive	Total
**0**	2	0	2
**1**	8	4	12
**2**	8	8	16
**3**	6	5	11
**Total**	**24**	**17**	**41**

*P*-Value 0.513278

**Table 2:** expression of IL-8RB in ovarian high grade serous carcinoma

	Resistant	Sensitive	Total
**0**	0	0	0
**1**	8	4	12
**2**	11	12	23
**3**	4	0	4
**Total**	**23**	**16**	**39**

*P* value 0.118982278

## DISCUSSION

We previously reported the expression of IL-8 and IL-8 receptors, IL-8RA and IL-8RB, in benign, borderline and malignant epithelial ovarian tumours of different histological subtypes. There was also a highly significant correlation between IL-8 and IL-8RA expression levels and tumour stage, being higher in tumours of higher stage and also with tumour type, with higher expression in serous carcinoma as compared to other histological types [21].

High grade serous carcinoma is the commonest type of ovarian cancer [22]. In this study we focused on investigating the functional role of IL-8 in platinum response in high grade serous carcinoma.

Links between resistance to paclitaxel and IL-8 in ovarian cancer cells have been previously reported. The paclitaxel-resistant ovarian cancer cell line, SKOV-3TR, showed overexpression of IL-8 [18]. Also paclitaxel can induce IL-8 gene expression at the transcriptional level by activating the IL-8 promoter in human ovarian cancer [19, 20].

Wang *et al* showed that treatment of non-IL-8-expressing A2780 ovarian cancer cells with exogenous recombinant IL-8 and overexpression of cellular IL-8 induce cisplatin and paclitaxel resistance, while depleting endogenous IL-8 expression in IL-8-overexpressing SKOV-3 cells using antisense IL-8 transfection promotes the sensitivity of the cells to drugs [23]. Their results suggest a similar link for cisplatin to IL-8 as has been previously reported for paclitaxel. However Domcke *et al* (2013) showed that A2780 and SKOV3 cells are very poor models of high grade serous ovarian cancer and therefore the relevance of IL-8 in the response to platinum in the common high grade serous subtype remains unanswered. The cell lines used in this study were derived directly from the malignant ascites or pleural effusions of high grade serous cancer patients treated at the Western General Hospital in Edinburgh, UK and are very well annotated for clinicopathological features and chemotherapy response (described in Cooke and Brenton (2010)).

In the present study we further explored this link using these intra-patient paired platinum sensitive and platinum resistant ovarian cancer cell lines that normally express IL-8 and IL-8 receptors. We show for the first time that in these clinically derived HGS cells the expression of IL-8 is enhanced by platinum treatment, and that this increased expression is consistent in all 4 studied cell lines, reaching a peak at 72 hours post treatment and then declining to baseline at 96 hours. Using immunocytochemistry we confirmed the expression of IL-8 receptors in all cell lines and show that the expression of receptors also increased on platinum treatment, with a change in subcellular localisation of IL-8RA into the nucleus. This is also the first study demonstrating that treatment with chemotherapeutic agents not only increases expression of IL-8, but also influences the level of expression and subcellular localisation of IL-8 receptors. The results show a significant link between treatment with cisplatin and enhancing IL-8 signalling activity through upregulation of expression of both ligand and receptors. The high expression of IL-8 in the presence of increased IL-8 receptor expression would set a circuit for autocrine and paracrine signalling [24], which is known to increase tumour cell growth, survival, migration and angiogenesis [25] and as we show platinum resistance and upregulation of anti-apoptotic proteins. These facts highlight IL-8 and IL-8 receptors as potential therapeutic targets in management of ovarian tumours.

Our results show that knocking down IL-8 by siRNA enhances sensitivity to platinum in the platinum sensitive cell line PEA1 and reverses platinum resistance in the platinum resistant cell line PEA2. This is demonstrated by increase in apoptosis as compared to controls represented by untreated cells or cells treated with non-targeting siRNA. Platinum resistance in PEA2 was also reversed using an IL-8 receptor blocking agent/antagonist. This provides promise for the clinical use of anti-IL-8 receptor agents in treatment of platinum resistant cases in conjunction with platinum based chemotherapeutic agents to improve drug sensitivity. Knowing the time line of the rise of IL-8 expression in HGS cells following platinum treatment as shown in this study helps making informed plans as to the optimal time of the concurrent administration of different agents.

It is well established that the effect of platinum on cells is principally mediated through its reaction with cellular DNA to cause damage, which finally triggers the cell's apoptotic pathway. Previous work in our lab has identified that platinum mediated DNA damage leads to a DNA-PKcs dependent activation of pro-survival AKT signalling, however this happens in platinum resistant cells only [26]. The data provided here indicates that the role of IL-8 in platinum response is independent of this mechanism as inhibition of IL-8 signalling appears equally effective at enhancing response in platinum sensitive and resistant cell lines, using the same cell line models as our previous study.

Changes in the level of expression or activity of apoptosis related proteins is one of the mechanisms of development of platinum resistance [27]. We hence explored the effect of knocking down IL-8 on the level of 2 anti-apoptotic proteins; bcl-2 and pBAD. Our results show the downregulation of both proteins following knock down of *IL-8*. The findings are in agreement with Wang *et al*. who demonstrated that the chemoresistance caused by IL-8 is associated with increased expression of apoptosis inhibitory proteins (Bcl-2, Bcl-xL, and XIAP) [23].

IL-8 mediates its biological effects via the two receptors, IL-8RA and IL-8RB. IL-8RA binds to granulocyte chemotactic protein-2 and IL-8 only. In contrast IL-8RB is more promiscuous and in addition to IL-8 is activated by several chemokines including Gro-α, β and γ, neutrophil activating peptide and granulocyte activation peptide-2 [28]. Our *in vitro* work showed that nuclear localisation of IL-8RA is precipitated by platinum treatment in the different cell lines. On studying tumour samples by IHC we found no correlation between the level of expression of IL-8 receptors and the status of platinum sensitivity of the tumours. However, nuclear IL-8RA expression was only found in platinum resistant tumours. The results suggest the detection of nuclear expression of IL-8 in an ovarian high grade serous carcinoma may be a potential marker for identifying a sub-population of platinum resistant tumours. The fact that cisplatin treatment increases expression of IL-8 and that decreased expression of IL-8 is associated with decrease and / or reversal of resistance suggest that it plays a role in resistance. Provisionally *in vitro* work shows that on increase of expression there is nuclear localisation. As increased expression *in vitro* is associated with nuclear localisation, it may be extrapolated that nuclear localisation can be a marker of resistance or decreased platinum sensitivity. In the small number of tumours studied, we only detected nuclear localisation in resistant cases. This is an initial observation suggesting that nuclear localisation of IL-8RA may be a potential biomarker for resistance. However, as the number of cases studied is small, a larger cohort with known response to platinum treatment needs to be tested to validate this observation.

In conclusion, our results show that IL-8 and IL-8 receptor expression by tumour cells increases on cisplatin treatment, and that IL-8 knockdown increases the cells’ sensitivity to cisplatin. The effect of IL-8 on platinum sensitivity appears to be mediated via an effect of IL-8 on anti-apoptotic proteins Bcl-2 and Bad. Our results form the basis for preclinical and clinical assessment of IL-8 and IL-8 receptors as therapeutic targets in conjunction with platinum based therapy.

## MATERIALS AND METHODS

### Cell lines and reagents

Intra-patient paired platinum sensitive and resistant high grade serous ovarian carcinoma cell lines (PEA1 vs PEA2 and PEO14 vs PEO23 respectively) were obtained from Dr. Simon Langdon (Edinburgh, UK) [29]. The cell lines PEA1 and PEO14) were derived prior to the onset of platinum resistance, whereas PEA2 and PEO23 were derived following acquired clinical platinum resistance and were cultured and maintained in RPMI-1640 medium, supplemented with 10% foetal calf serum, 1% Penicillin Streptomycin and L-Glutamine (all GIBCO, UK) at 37°C/5% CO_2_.

Antibodies used were IL-8-RA/CXCR1 and IL-8-RB/CXCR2 (R&D systems, UK); Alexa Fluor 594 (BD Diagnostics, UK); Bcl-2 and p-Bad S136 (Cell Signalling Technology, UK); HSP60 (Abcam, UK); goat anti-mouse and goat anti-rabbit HRP conjugated secondary antibodies (DAKO, Denmark). Blocking of IL-8-RA/B Signalling was achieved using a small molecule antagonist SCH563705 [30, 31].

### Assessment of IL-8 expression during continuous exposure to platinum

Matched pairs of platinum sensitive or resistant cells were seeded into 6 well plates and incubated for 24 hours. Platinum sensitive or resistant cells were treated with cisplatin at doses relative to their previously determined IC50 concentrations (final concentration 2.5 μM or 12.5 μM cisplatin respectively) and incubated for 0, 24, 48, 72 and 96 hours. RNA was prepared from cells at each time point using RNeasy mini kit according to manufacturer's protocol (Qiagen, UK). *IL-8* levels were determined by qRT-PCR as described below.

### siRNA transfection, apoptosis and cell viability assays

The matched cell line pairs, PEA1/PEA2 and PEO14/PEO23, were transfected with siRNAs directed to IL-8 or non-targeting control 2 (NT2) (GE Dharmacon, USA) at 60% confluency and re-transfected 48 hours later. Cells were transfected with siRNAs at final concentration of 100 nM using DharmaFECT 1 transfection reagent (GE Dharmacon, USA) according to manufacturer's instructions. Twenty-four hours after second transfection, cells were reseeded into 6 well plates and incubated for 48 hours. Cell lysates were collected and RNA extracted using RNeasy mini kit according to manufacturer's protocol (Qiagen, UK). Caspase activation was measured using Caspase 3/7 Glo assay (Promega, UK) and activity normalised to cell viability inferred by MTT assay, performed as described elsewhere [26, 32].

### Reverse transcription and determination of gene expression using quantitative real-time PCR

cDNA was synthesised using 1 μg of total RNA, 1 μl oligodT primer (0.5 μg/μl) (Life Technologies, UK) 1 μl RNasin, 4 μl 5x MMLV buffer, 1 μl 10 mM dNTP mix and 0.3 μl of MMLV reverse transciptase (Promega, UK) made up to a final volume of 20 μl with RNase free water. Reactions were incubated at; 65°C for 10 minutes and chilled to 4°C for 5 minutes. Reactions were then incubated for a further hour at 37°C, stopped by heating to 95°C for 5 minutes, then cooled to 4°C.

Real time PCR master mix consisted of; 5 μl Faststart Universal SYBR Green Master (Rox) (Roche, UK), 1 μl of each forward and reverse primers at 12.5 μM (Life Technologies, UK), 1μl RNase free water, 2 μl cDNA. PCR cycling parameters were as follows; 95°C 10 mins, 95°C 10 secs, 60°C 30 secs for 40 cycles and run on 7900HT Fast Real-Time PCR System (Applied Biosystems). Gene expression data was normalised in all reactions to its endogenous peptidylprolyl isomerise A (PPIAs) control. mRNA levels of cisplatin treated cells were normalised to untreated cells to obtain the net value in mRNA expression after platinum treatment. Primer sequences were as follows: PPIA Forward- 5′-TGCTGTATTGTTGCCCATGT-3′; PPIA Reverse-5′-GATCAAATCCGCCACCTCTA-3′; IL-8 Forward – 5′-CTTGTCATTGCCAGCTGTGT-3′; IL-8 Reverse – 5′-TGACTGTGGAGTTTTGGCTG-3′.

### Blocking of IL-8 signalling using IL8RA/RB antagonist

The IL-8RA/RB antagonist SCH563705 [30, 31] was used at a final concentration of 100 nM. Matched platinum sensitive and resistant cell lines PEA1 and PEA2 were seeded at a density of 1 × 10^4^ per well in 96 well plates or 2 × 10^5^ per well in 6 well plates and treated with the antagonist alone and/or cisplatin (10 μM (PEA1) or 25 μM (PEA2)) for 24 hours. Apoptosis and cell viability assays were carried out as described above. Cell lysates were prepared for both RNA and protein extraction.

### Determination of pro-apoptotic protein levels by Western blotting

Cells subjected to IL-8 siRNA were harvested and lysed in radioimmunoprecipitation (RIPA) assay buffer (Santa Cruz Biotechnology, USA) including 1% phosphatase inhibitor, protease inhibitor and sodium orthovanadate. Protein lysates were run on SDS-polyacrylamide gels and blotted onto nitrocellulose membrane (Pall Corporation, USA). Following transfer, membranes were blocked in 5% bovine serum albumin (BSA) in TBS-Tween, probed with antibodies for HSP60, Bcl-2 or p-Bad S136 at a dilution of 1:1000 in 5% BSA/TBS-T overnight at 4°C, washed three times in TBS-T, incubated in corresponding secondary antibodies for 1 hour at room temperature, washed three times, and bound antibodies visualized by Immobilon enhanced chemiluminesence (Millipore, UK).

### Immunofluorescence and confocal microscopy

Cells were grown on coverslips for 24 hours at a concentration of 1 × 10^4^ for PEO1, PEA2, PEO14, PEO23 cells, at 3 × 10^4^ for PEO4 and 9 × 10^3^ for PEA2 cells respectively. Cells were treated with either cisplatin for 24 hours at a concentration of 5 μM for platinum sensitive and 25 μM for platinum resistant cells or media only (untreated). Cells were fixed with 4% paraformaldehyde for 30 min at 37°C and rendered permeable in 0.2% triton. Coverslips were washed with PBS and blocked in 2% FCS/1% BSA solution for 30 min at 37°C. Cells were incubated with the primary antibody for IL-8RA (1:2000) or IL-8RB (1:200) for 1 hour at 37°C. After rinsing in PBS, the cells were incubated in the secondary antibody Alexa Fluor 594 (1:600) for 1 hour at 37°C. For negative control experiments, cells were similarly treated with the primary antibody omitted. Cover slips were affixed to slides with mounting media containing DNA-binding DAPI dye (Vectashield, Vector Labs, UK). Cells were examined with a Zeiss LSM 510 confocal microscope, using an x63 oil immersion lens.

### Immunohistochemistry for IL-8RA and IL-8RB expression in platinum sensitive and resistant ovarian tumours

Forty-one ovarian tumours, all high grade serous carcinoma, were studied. Seventeen tumours were from confirmed platinum sensitive and 24 from platinum resistant patients who underwent surgery and received treatment at the Hammersmith Hospital, London, United Kingdom. Tumour specimens were available as formalin fixed, paraffin embedded tissue blocks. All slides were reviewed and representative blocks of the tumours were selected for the study. Ethics committee approval was obtained from Hammersmith and Queen Charlotte's and Chelsea Research Ethics Committee (REC reference: 05/Q0406/178).

The expression of IL-8RA and IL-8RB was investigated using an indirect two-stage method. Tissue sections were de-waxed and rehydrated by passing the slides through xylene and descending grades of alcohol then water. Slides were then incubated for 15 minutes with 0.6% hydrogen peroxide solution to block endogenous peroxidase activity, followed by heat-mediated antigen retrieval, using 0.1M citrate buffer pH 6.0 and microwaving for 20 minutes. Slides were then attached to a sequenza racks. To block non-specific binding 100 μl of Protein Block were added to each slide for 5 minutes and the slides were incubated with 100 μl of the corresponding primary antibody (1:8000 for IL-8RA and 1:400 for IL-8RB) at 4°C overnight.

Slides were washed with 0.05% PBS/Tween 20 solution, followed by 5 minutes incubation with 100 μl of post primary block. After another wash, the slides were developed with the addition of 100 μl 3, 3′-Diaminobenzidine (DAB) solution followed by counterstaining with haematoxylin.

Slides were then dehydrated in ascending grades of alcohol, cleared with xylene and mounted using Di-N-Butyle Phthalate in Xylene (DPX) mountant and covered with a glass coverslip.

Sections of appendix tissue with acute appendicitis were used as a positive control in each run of staining. Additionally, the presence of inflammatory cells within the test sections acted as further internal positive controls. For negative controls, duplicate slides from each case were used, and incubated with 100 μl antibody diluent instead of primary antibody.

The stained slides were then examined by light microscopy to assess the expression and subcellular distribution of IL-8RA and IL-8RB. The level of expression was scored as weak (1), moderate (2) or strong (3).

### Statistical analysis

Statistical analysis of normalised caspase activity and gene expression analysis was performed using GraphPad Prism^®^ version 5.03. The data represented is mean ± standard deviation (SD) or mean ± standard error mean (SEM) as indicated. Statistical analysis was performed using an upaired, two tailed student's *T*-test.

The Chi-square test was used to test for the presence of correlations between antigen expression in tumours and response to platinum. Significant correlation was considered with *p* ≤ 0.05. Statistical analysis was performed using SPSS (version 16.0, Chicago, IL, USA).

## SUPPLEMENTARY FIGURE


