# Supplementary material for: The role of interleukin-8 (IL-8) and IL-8 receptors in platinum response in high grade serous ovarian carcinoma
Source: Oncotarget. 2015 Mar 23;6(31):31593–603. doi: 10.18632/oncotarget.3415 (PMC4741626; doi:10.18632/oncotarget.3415)
Supplement: Supplementary file 1 [file oncotarget-06-31593-s001.pdf]

## SUPPLEMENTARY FIGURE

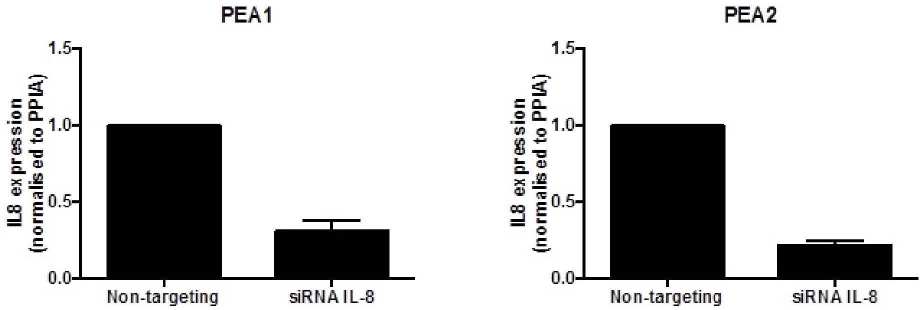


**Supplementary Figure S1: siRNA for IL-8.** Knockdown is confirmed using IL-8 siRNA in comparison to non-targeting control and untreated.
